# Collagenase-producing bacteria are common in anastomotic leakage after colorectal surgery: a systematic review

**DOI:** 10.1007/s00384-023-04562-y

**Published:** 2023-12-01

**Authors:** Anders Bech Jørgensen, Isabella Jonsson, Lennart Friis-Hansen, Birgitte Brandstrup

**Affiliations:** 1 Department of Surgery, Holbæk Hospital, Part of Copenhagen University Hospitals, Region Zealand, Denmark; 2 Department of Clinical Medicine, Faculty of Health and Medical Sciences, University of Copenhagen, Copenhagen, Denmark; 3 Department of Clinical Biochemistry, Bispebjerg and Frederiksberg University Hospital, Capital Region, Frederiksberg, Denmark; 4 Department of Microbiology, Rigshospitalet, Capital Region Denmark

**Keywords:** Anastomotic leakage, Colorectal surgery, Bacterial collagenase, Gut microbiome, Complications

## Abstract

**Purpose:**

Some gut bacteria can produce enzymes (collagenases) that can break down collagen in the intestinal wall. This could be a part of the pathophysiology of anastomotic leakage (AL). This systematic review aimed to investigate if such bacteria were present more frequently in AL patients versus non-AL patients following colorectal surgery.

**Methods:**

This systematic review was reported according to the PRISMA and AMSTAR guidelines. Before the literature search, a study protocol was registered at PROSPERO (CRD42022363454). We searched PubMed, EMBASE, Google Scholar, and Cochrane CENTRAL on April 9^th^, 2023, for randomized and observational human studies of AL following colorectal surgery with information on gastrointestinal bacteria. The primary outcome was bacteria with the potential to produce collagenase. The risk of bias was assessed with the Newcastle–Ottawa Scale, as all studies were observational.

**Results:**

We included 15 studies, with a total of 52,945 patients, of which 1,747 had AL, and bacteriological information from feces, mucosa, the resected specimen, or drain fluid was presented. In 10 of the 15 studies, one or more collagenase-producing bacteria were identified in the patients with AL. Neither the bacteria nor the collagenase production were quantified in any of the studies. The studies varied greatly in terms of sample material, analytical method, and time of collection. Studies using DNA sequencing methods did not report findings of collagenase-producing bacteria.

**Conclusion:**

Collagenase-producing bacteria are more common in patients with AL following colorectal surgery than in patients without AL, but the significance is unclear. From the current studies, it is not possible to determine the pathogenicity of the individual gut bacteria.

**Supplementary Information:**

The online version contains supplementary material available at 10.1007/s00384-023-04562-y.

## Introduction

Surgical resections of parts of the colon and rectum are common procedures for removing benign conditions (e.g., inflammatory bowel or diverticular diseases), precancerous lesions, or malignant tumors. In most patients, it is possible to re-establish gut continuity with an anastomosis, and the surgical wound in the intestine usually heals within 7–10 days. However, a major complication is anastomotic leakage (AL), which occurs in 2–15% of the patients, with the occurrence rate depending on the specific location of the anastomosis [1–6]. Anastomoses in the right side of the colon have the lowest risk of AL (2–5%), and anastomoses to the rectum have the greatest risk of AL (10–15%). Among other risk factors for AL are male gender, increasing age, American Society of Anesthesiologists (ASA) classification > II, renal disease, smoking, obesity, poor nutrition, neoadjuvant chemoradiotherapy, blood loss/transfusion during surgery, and duration of surgery [3, 7–9]. AL is associated with considerably increased morbidity, reduced quality of life, prolonged hospital stay (including stays in the intensive care unit) [10], increased costs [11], increased risk of recurrence of cancer, and increased risk of death [12].

Collagen is one of the key components of the extracellular matrix (ECM), and the formation and deposits of collagen fibers are essential for the normal strength of the intestinal wall [13]. However, the surgery disrupts the ECM, causing reduced tensile strength of the collagen fibers in the submucosal layer around the suture material [14].

Healing of the surgical wound is a complex process that can roughly be divided into four partly overlapping temporal phases: Hemostasis, inflammation, proliferation (cellular infiltration, angiogenesis, and re-epithelialization), and maturation/remodeling [15]. Collagen plays a pivotal role in regulating these phases [16]. Disruption of any of the wound healing phases will fixate the healing process in a chronic, non-healing state. Persistent inflammation can lead to chronic wounds by elevating matrix metalloproteinases (MMP) levels and other enzymes. This increases the destruction of ECM components and improper activation of soluble mediators of the wound healing process. Not only is the ECM modified by MMPs and other endogenous enzymes originating from the cells within the wound, but the ECM can also be affected by exogenous enzymes from the bacteria in the gut.

One major group of these is the collagenases, a group of enzymes belonging to the metalloproteinase family, which can play a significant role in the degradation of extracellular collagen fibers. The collagenase enzyme consists of three chains of repeating amino acids that together form a triple helix structure. It has two primary units: the collagen-binding and catalytic domains [17]. The remodeling process of collagen within the intestinal wall is influenced by collagenases originating from either cells that are part of the wound healing process or bacteria that use these as part of their mechanism to invade the human organism [18, 19]. These collagenase-producing bacteria are classified by The Nomenclature Committee of the International Union of Biochemistry and Molecular Biology (NC-IUBMB) under the EC 3.4.24.3 group. Still, more than 50 other bacteria have been reported to have collagenolytic activity (presented in Online Resource 1). The most clinically relevant bacteria are *Enterococcus faecalis* [18, 20], *Clostridioides difficile* [21], *Klebsiella pneumoniae* [22], *Pseudomonas aeruginosa* [19, 23, 24], *Proteus mirabilis* [24, 25], and *Bacteroides* spp. [24, 26, 27].

The idea that there might be a causal relationship between gut bacteria and AL was supported by a recent study [28], where feces from patients with AL after colorectal surgery were transferred to mice undergoing colorectal surgery. The mice showed poorer colonic healing, including reduced levels of ECM components in the wound and increased collagen degradation activity.

The aim of this systematic review was to investigate if bacteria capable of producing collagenase were present more frequently among AL patients vs. non-AL patients following colorectal surgery.

## Methods

This systematic review follows the PRISMA (Preferred Reporting Items or Systematic Reviews and Meta-Analysis 2020) [29] and AMSTAR (Assessing the Methodological Quality of Systematic Reviews) guidelines [30]. The PRISMA checklist is available in Online Resource 2.

The protocol was registered at PROSPERO (International Prospective Register of Systematic Reviews) before the literature search (registration number CRD42022363454). Originally, the intention was to present both animal and human studies on the topic. Therefore, our study is registered and approved with PROSPERO as an “Animal research study” with additional information on how to handle human studies. Later in the review process, it was decided only to include human studies. Changing the type of PROSPERO registration from “Animal research study” to “Health research study” was not possible.

### Eligibility criteria

The literature search followed the principles of PICO (Patients, Intervention, Comparator, Outcome). The participants were patients with previous colorectal resection and anastomosis, where the studies presented data of bacteria/gut microbiome in either feces, mucosa, the resected specimen, or drain fluid. AL was the exposure (intervention), and similar patients without AL were the comparator.

The primary outcome was identifying bacteria with the potential to produce collagenase; the secondary outcome was reports of other bacteria found.

We included all randomized and observational studies written in English or Danish. There was no restriction on publication year.

### Information sources and search strategy

The literature search was conducted on November 30, 2022, and updated on April 8, 2023. We searched PubMed (from 1946 to present), EMBASE (OVID interface, from 1974 to present), Google Scholar (from 2004 to present), and Cochrane Central Register of Controlled Trials (CENTRAL) (from 1992 to present).

The principle of the literature search was a bloc search strategy. Three blocks should cover the topics of AL, colorectal surgery, and bacterial collagenase. The search string for PubMed was: (“anastomotic leak”[MeSH Terms] OR “anastomotic leak*”[All Fields] OR “insufficient anastomosis”[All Fields] OR “anastomotic insufficiency”[All Fields] OR “anastomotic dehiscence”[All Fields] OR “leak*”[All Fields]) AND (“colorectal surgery”[MeSH Terms] OR “colon”[All Fields] OR “colonic”[All Fields] OR “colorectal”[All Fields] OR “rectal”[All Fields] OR “ileorectal”[All Fields] OR “ileoanal”[All Fields] OR “colorectal surgery”[All Fields] OR “colorectal resection”[All Fields] OR “colorectal anastomosis”[All Fields] OR “colon surgery”[All Fields] OR “colon resection”[All Fields] OR “colon anastomosis”[All Fields] OR “colonic surgery”[All Fields] OR “colonic resection”[All Fields] OR “colonic anastomosis”[All Fields] OR “rectal surgery”[All Fields] OR “rectal resection”[All Fields] OR “rectal anastomosis”[All Fields] OR “ileorectal anastomosis”[All Fields] OR “ileoanal anastomosis”[All Fields]) AND (“microbial collagenase”[MeSH Terms] OR “collagenase”[All Fields] OR “collagenoly*”[All Fields] OR “bacterial collagenase”[All Fields] OR “microbial collagenase”[All Fields] OR (“enterococcus”[MeSH Terms] OR “enterococcus”[All Fields]) OR “Pseudomonas aeruginosa”[All Fields] OR “Proteus mirabilis”[All Fields] OR “Bacteroides fragilis”[All Fields] OR “16S”[All Fields] OR “culture”[All Fields] OR “sequenc*”[All Fields]). The search strategies for EMBASE, Google Scholar, and Cochrane CENTRAL are included as Online Resource 3, along with an overview of the bloc search strategy.

In addition to these searches, we performed a snowball search of the reference lists of the included studies, to identify additional studies that could meet the inclusion criteria. To retrieve missing data, we contacted the authors by e-mail.

### Study selection

The search results were imported into the online review management solution Covidence [31], automatically removing duplicates. One of the authors verified that no papers were removed by mistake. Two authors screened all the abstracts, and any disagreements were solved by conference with a third author. We excluded case reports, conference abstracts, animal studies, and studies that had not been peer-reviewed.

### Data collection

We extracted data from the included studies to a spreadsheet prepared in advance. Author information, year of study, study design, sample material (feces, drain fluid, perioperative sample, and mucosal swab/tissue), method (culture, next-generation sequencing (NGS), quantitative polymerase chain reaction (qPCR), or *Clostridioides difficile* immunochemical assay (CDI)), patient demography (number of patients, gender, antibiotic usage, bowel preparation, indication for surgery, type of surgery, anastomotic technique, diverting ileostomy, and usage of drain), and outcome (AL-rate, information on any bacteria able to produce collagenase, and other bacteria) were extracted from the studies. Relevant statistical measures were also noted.

### Risk of bias

Two authors independently assessed the risk of bias for the studies according to study type. We used Cochrane Risk of Bias tools for randomized trials (Rob 2) [32]. For observational or case–control studies, we used the Newcastle–Ottawa scale (NOS) [33]. However, we made the following minor modifications: For cohort and case–control studies, each study can be graded with up to nine stars combined from three categories (Selection, Comparability, and Outcome). As the “Non-exposed cohort” in the cohort studies obviously had to come from the same group of patients as the “Exposed,” this point in the “Selection” section was left out. Similarly, the “Selection of controls” point was left out in the “Selection” section of the case–control studies because all studies used hospital controls. Therefore, cohort and case–control studies could be assigned a maximum of eight stars. The higher the number of stars, the lower the risk of bias.

The outcome of our study is a description of the bacteria found in the case of AL. Under the “Comparability” category, we awarded each study one star in 1a if the study distinguished colonic from rectal AL and if bacterial information was presented separately for each group. Likewise, in 1b, a star was only awarded if the study presented other relevant risk factors for AL (for instance gender, age, smoker status, or ASA classification) along with specified bacterial information. Any doubts about the grading were solved by conference with the senior author.

### Data synthesis and statistics

We planned to do a descriptive presentation of the studies as we anticipated finding heterogeneous studies in a limited number. As both primary and secondary outcomes were reports of the association between the bacteria found in feces/mucosa/specimen/drain fluid in patients with anastomotic leakage, we did not plan to conduct a meta-analysis.

## Results

**Fig. 1:**
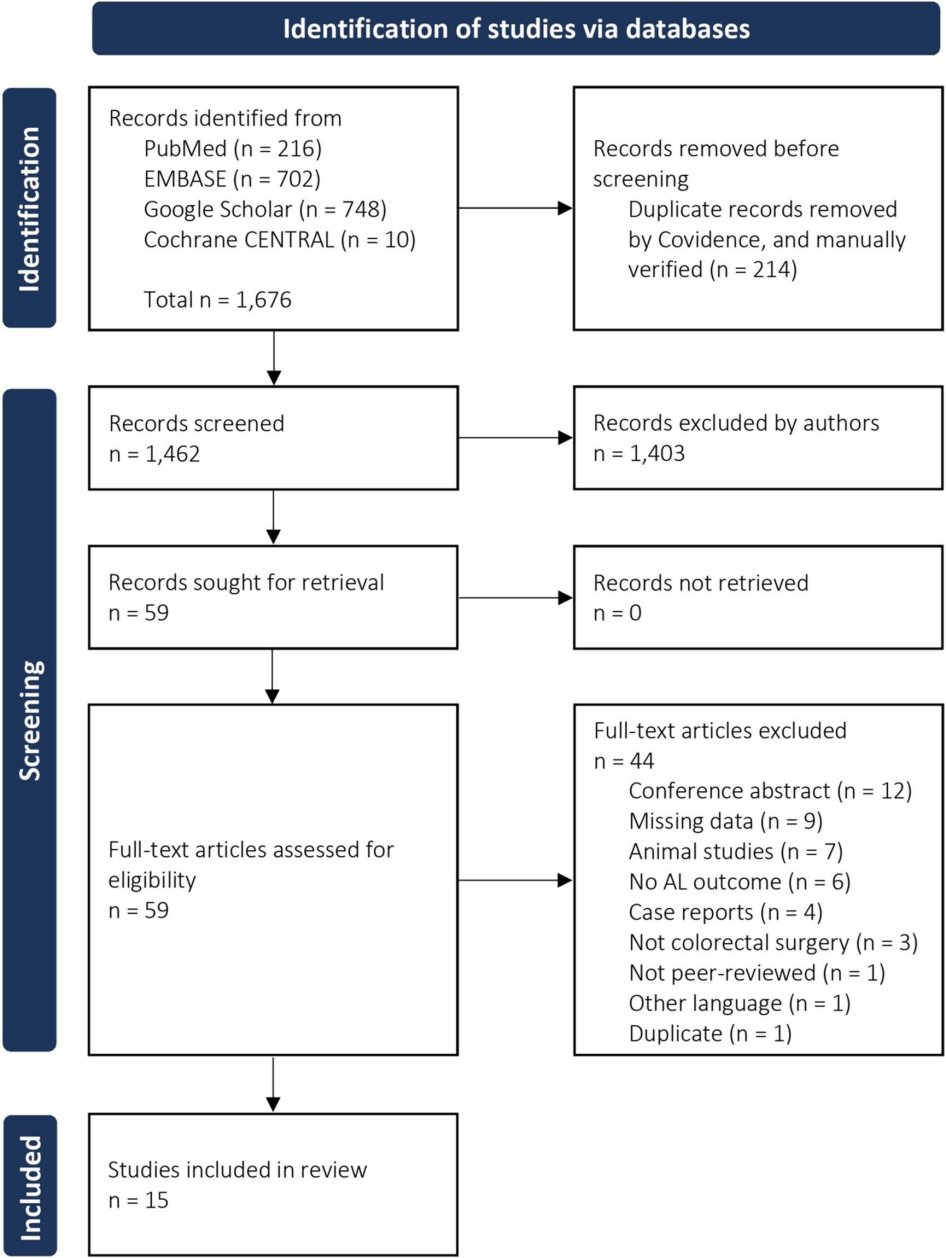
PRISMA flow diagram for study screening and selection. PRISMA, Preferred Reporting Items for Systematic Reviews and Meta-Analyses

We found a total of 1,676 papers in the search. There were 216 from PubMed, 702 from EMBASE, 748 from Google Scholar, and ten from Cochrane CENTRAL. Out of these, 214 papers were marked as duplicates by Covidence, and this was manually verified. We screened 1,462 abstracts for inclusion, and 1,403 did not meet the inclusion criteria. For the remaining 59 studies, it was possible to retrieve all records for full-text assessment, and of 59 articles, we excluded 44 due to various reasons (12 conference abstracts, nine had no relevant data, seven were animal studies, six had wrong outcome, four were case reports, three were not colorectal surgery, one duplicate, one not peer-reviewed, and one was written in another language than Danish or English). We included 15 studies in this systematic review [34–48]. Figure 1 presents the PRISMA 2020 flow diagram for study selection.

### Study characteristics

Most studies (80%) were published from 2019 onwards [34, 35, 37, 39, 41–48]. The majority of the studies were case–control studies [35, 36, 38–41, 43–48], and three were descriptive studies [34, 37, 42].

The number of patients varied greatly from as few as 19 patients [34] to more than 46,000 patients [35]. The total number of patients in all studies was 52,945, of which 1,747 had AL. All studies except one [35] had a majority of male patients. However, one study did not report the sex of the patients [42]. The rate of AL was reported in 14 out of 15 studies [35–48]. The use of antibiotics was reported in nine studies [35–38, 40, 43, 45, 46, 48], and information about bowel preparation was presented in eight studies [35–38, 40, 43–45]. Less than half of the studies reported the type (hand-sewn or stapled) of anastomosis constructed [36–38, 40, 46–48]. The formation of a diverting ileostomy at the primary operation was noted in eight studies [37, 38, 40, 42, 43, 46–48], along with the placement of a drain [36, 40–43, 46–48]. These parameters had a lot of missing values but are still presented as they are important factors in assessing the quality of the surgery and the anastomosis. The indication for surgery was presented in all studies [34–48]. There were 11 studies on colorectal cancer patients [36–39, 41–47], and four studies were a mixture of benign and malignant colorectal diseases [34, 35, 40, 48]. One study reported only patients with occult AL [47], a small leak from the anastomosis without any clinical findings. The study characteristics are presented in Table 1.

**Table 1:** Study characteristics

**Author**	**Year**	**Study design**	**No. of** **patients**	**Men (%)**	**No. of AL (%)**	**Indication for surgery**	**Bowel preparation**	**Antibiotics**	**Diverting ileostomy**	**Drain**
Anderson et al. [34]	2021	Descriptive	19	10 (53)	19 (100)	IBD, cancer, other	-	-	-	-
Baker et al. [35]	2022	Case–control	46,735	22,354 (48)	1,449 (3)	Diverticular disease, cancer, IBD, bleeding, perforation, other	NS	NS	-	-
Bilgin et al. [36]	2017	Case–control	50	35 (70)	7 (14)	Rectal cancer	Oral, enema	IV	-	X
Calu et al. [37]	2019	Descriptive	320	181 (57)	19 (6)	Colorectal cancer	Oral	Oral, IV	(X)	-
Fouda et al. [38]	2011	Case–control	56	40 (71)	8 (14)	Rectal cancer	Oral	IV	(X)	-
Jin et al. [39]	2022	Case–control	34	19 (56)	17 (50)	Colorectal cancer				
Komen et al. [40]	2014	Case–control	243	135 (54)	19 (8)	Colorectal cancer, IBD, ischemic colitis	NS	NS	(X)	X
Li et al. [41]	2019	Case–control	37	19 (51)	7 (19)	Colorectal cancer	-	-	-	(X)
Lohsiriwat and Assawasirisin [42]	2020	Descriptive	4,357	NA	84 (2)	Colorectal cancer	-	-	(X)	(X)
Mima et al. [43]	2020	Case–control	256	152 (59)	29 (11)	Colorectal cancer	Oral	IV	(X)	(X)
Palmisano et al. [44]	2020	Case–control	21	11 (52)	5 (24)	Colorectal cancer	Oral	-	-	-
Reuvers et al. [45]	2022	Case–control	223	136 (61)	20 (9)	Colorectal cancer	Oral^a^	IV, SDD	-	-
Sparreboom et al. [46]	2019	Case–control	292	193 (66)	38 (13)	Colorectal cancer	-	Oral	(X)	X
Tominaga et al. [47]	2022	Case–control	243	126 (52)	16 (7)	Colorectal cancer	-	-	(X)	X
van Praagh et al. [48]^b^	2019	Case–control	59	45 (76)	10 (17)	Colorectal cancer, diverticular disease, other	-	Oral	(X)	(X)

*AL* anastomotic leakage, *IBD* inflammatory bowel disease, *NS* not specified (the use of bowel preparation and antibiotic administration is stated, but not specified), *IV* intravenous, *(X)* drain/diverting ileostomy present in some but not all patients, *NA* not available, *SDD* selective decontamination of the digestive tract (the patients were randomized to either standard intravenous antibiotic prophylaxis (control group) or standard intravenous antibiotic prophylaxis plus per oral colistin, tobramycin, and amphotericin B for three days before surgery)

^a^Only for left-sided or rectal resections

^b^This study was a substudy of the C-seal trial [49], in which half of the patients had an intraluminal biodegradable soft sheath (C-seal) stapled to the anastomosis (only the patients without the C-seal were included in this review)

### Sample material and study methods

The majority of studies reported drain fluid as the sample material [34, 36, 38, 40, 46, 47]. Fecal samples were reported in four studies [35, 37, 39, 44], perioperative intraabdominal sampling in two studies [34, 42], mucosal tissue in three studies [41, 43, 48], and one study reported rectal mucosal swabs as sample material [45].

The composition of bacteria in the gut, feces, or abdomen was primarily characterized by cultures [34, 36, 38, 42, 47]. DNA sequencing methods were used in five studies [39, 41, 44, 45, 48], although one study did not specifically report exactly which part of the bacterial DNA had been sequenced [44]. Other study methods were qPCR in three studies [40, 43, 46] and enzyme immunoassay for *Clostridioides difficile* infection (CDI) in two studies [35, 37]. One study used visual identification and counting with Gram staining of bacteria as the study method [47]. An overview of sample material, time of collection, and study methods is presented in Table 2.

**Table 2:** Sample material, time of collection, and study methods

**Author**	**Year**	**Sample material**				**Time of collection**	**Methods**			
		**Feces**	**Drain**	**Mucosa**	**Abdomen**		**Culture**	**NGS**	**qPCR**	**CDI**
Anderson et al. [34]	2021		X		X^a^	Peri/Post	X			
Baker et al. [35]	2022	X				Post				X
Bilgin et al. [36]	2017		X			Post	X			
Calu et al. [37]	2019	X				Post				X
Fouda et al. [38]	2011		X			Post	X			
Jin et al. [39]	2022	X^b^				Post		X		
Komen et al. [40]	2014		X			Post			X	
Li et al. [41]	2019			X^c^		Peri		X		
Lohsiriwat et al. [42]	2020				X^d^	Peri	X			
Mima et al. [43]	2020			X^e^		Peri			X	
Palmisano et al. [44]	2020	X^f^				Pre		X		
Reuvers et al. [45]	2022			X^g^		Peri		X		
Sparreboom et al. [46]	2019		X			Post			X	
Tominaga et al. [47]	2022		X			Post	X			
van Praagh et al. [48]	2019			X^h^		Peri		X		

*Pre* preoperative, *Peri* perioperative, *Post* postoperative, *NGS* next-generation sequencing, *qPCR* quantitative polymerase chain reaction, *CDI Clostridioides difficile* immunochemical assay

^a^Sample material not specified

^b^From the first postoperative defecation

^c^Adjacent to the surgical margin

^d^From fluid/abscess/debris

^e^Both from colorectal carcinoma tissue and 5–10 from the edge of tumor

^f^At the time of the first medical examination

^g^Perioperative rectal swab

^h^From donut tissue after circular stapling

### Case–control group comparison

Twelve studies were case–control studies [35, 36, 38–41, 43–48], in which cases of AL were compared with controls without AL. One study found that the risk of AL had an increased OR of 2.34 among patients with CDI [35]. A similar finding was that the risk was increased almost fourfold among patients with increased levels of *Bifidobacterium* spp. in mucosal tissue adjacent to the anastomosis [43]. DNA sequencing of preoperative fecal samples showed that two specific bacteria (*Acinetobacter lwoffii* and *Acinetobacter johnsonii*) were found exclusively among AL patients, whereas non-AL patients had *Barnesiella intestinihominis* (absent among AL patients) [44]. Infectious complications (including AL) were reduced with diminished levels of *Proteobacteria* [45] from rectal swabs, and there was increased alpha diversity in postoperatively collected fecal samples from AL patients [39]. Another study found increased levels of the families *Lachnospiraceae* and *Bacteroidaceae* in mucosal tissue among AL patients, and they presented a prediction model for AL based on this in combination with the alpha diversity of the samples [48].

Cultures from drain samples showed that almost all AL patients had positive cultures on postoperative day (POD) 5 [36], whereas nearly all non-AL patients had a negative culture [38]. One study showed no difference in positive cultures among AL and non-AL patients on POD1-3 [46], and another found that the absence of *E. faecalis* in drain fluid on POD3 had a high negative predictive value for AL [40]. The identified bacteria in all studies and the type of surgery, are presented in Table 3.

**Table 3:** Bacterial findings

**Author**	**Type of surgery**	**Collagenase bacteria**	**Other results**	**Comments**
Anderson et al. [34]	Colon or rectal resection	Present in 73.7% of AL patients *E. faecalis* present in 36.8% of AL patients	-	-
Baker et al. [35]	Colon or rectal resection	*Clostridioides difficile*	AL with CDI 3.52% AL without CDI 1.44%	CDI increases risk of AL (OR 2.39; 95%CI 1.70–3.36; *p* < 0.001)
Bilgin et al. [36]	Rectal resection	*Proteus* spp. and *Klebsiella pneumoniae* were found only in AL patients	AL patients culture-positive on POD1 (28.6%), POD3 (42.9%), and POD5 (85.7%), compared with 9.3%, 7%, and 11.6% in non-AL	All AL occurred in patients with stapled anastomosis (*p* = 0.325)
Calu et al. [37]	Colon or rectal resection, or colectomy	*Clostridium difficile*	AL in 68.4% who developed CDI after surgery AL was associated with CDI (RR 13.7; 95%CI 7.68–24.5; *p* < 0.0001)	AL patients all had left-sided or rectal resection
Fouda et al. [38]	Rectal resection	*E. coli* ↑*, Klebsiella* ↑*, Bacteroides* ↑ and *Pseudomonas* spp. ↑ in AL vs. non-AL group	85% of non-AL patients had negative cultures, and the remaining 7 only limited growth of *E. coli, Klebsiella* and *Pseudomonas*	-
Jin et al. [39]	Colon resection	*Bacteroides* spp.	AL-patients: *Romboustia, Blautia*, *Bacteroides and Eggerthella* ↑, *Clostridium *sensu stricto* 1* ↑*, Ruminococcus gnavus* ↑ Non-AL patients: *Lactobacillus* and *Comamonas* genera both ↑	Alpha-diversity of postoperative fecal samples was significantly higher in the AL group vs. non-AL group
Komen et al. [40]	Colon or rectal resection, or colectomy	*E. faecalis* ↑ on POD2-4 *E. coli* ↑ on POD4-5	No presence of *E. faecalis* on POD3, had a high negative predictive value (99%) and sensitivity (93%) for later AL	False positive results could be due to subclinical AL
Li et al. [41]	Colon or rectal resection	No	AL-group: *Corynebacterium suicordis, Porphyromonas asaccharolytica, Vibrio diazotrophicus,* and *Clostridium leptum* all ↑ Non-AL group: *Alistipes shahii* and *Dialister pneumosintes* were both ↑, and not found in the AL group	Notably, 4 out of 7 cases of AL were from right-sided anastomosis
Lohsiriwat et al. [42]	Colon or rectal resection, or colectomy	*Enterococcus*, *Pseudomonas* and *Klebsiella* spp.	Of 55 cultures from AL patients; 51 cultures showed 1 or more bacteria	-
Mima et al. [43]	Colon or rectal resection	Increased risk of AL with increased amounts of *Bifidobacterium* (OR 3.96, 95% CI 1.50–10.51)	Association between *Fusobacterium nucleatum, E. coli,* or *E. faecalis* with AL was not significant	-
Palmisano et al. [44]	Colon or rectal resection	No	AL-patients: *Acinetobacter lwoffii* and *Acinetobacter johnsonii* present (absent in non-AL patients) *Hafnia alvei* ↑, *Faecalibacterium prausnitzii* ↓ Non-AL patients: *Barnesiella intestinihominis* present (absent in AL patients)	Three species (*Anaerostipes caccae, Clostridium clariflavum,* and *Roseburia inulinivorans*) were found in males, but practically absent in females
Reuvers et al. [45]	Colon or rectal resection	No	Infectious complications were reduced with *Proteobacteria* ↓	
Sparreboom et al. [46]	Rectal resection	No difference in *E. coli* or *E. faecalis* levels on POD1-3 in drainage fluid among AL vs. non-AL patients	-	AL with or without diverting ileostomy (11.4% vs. 14.9%; *p* = 0.371)
Tominaga et al. [47]	Colon or rectal resection	Occult leakage: *P. aeruginosa* (28.6%), *E. faecalis* (42.9%) Culture-positive: *Enterobacter cloacae* (11.3%)	Occult leakage: *Enterobacter cloacae* (50%) Culture-positive: *P. aeruginosa* (21.0%), *E. faecalis* (19.4%)	Drain fluid on POD6 was used for Gram staining (GS) and culture GS-positive patients had a pelvic CT-scan
van Praagh et al. [48]	Colon or rectal resection	No	AL vs. non-AL group: *Lachnospiraceae* (40% vs. 27%; *p* = 0.010), *Bacteroidaceae* (28% vs. 17%; *p* = 0.008), and *Blautia obeum* (7% vs. 3%; *p* = 0.005) Presence of *Prevotella copri*, *Streptococcus* genus, and *Eubacterium biforme* was associated with a reduced risk of AL	The study presented a model for the prediction of AL, based on levels of *Lachnospiraceae*, *Bacteroidaceae,* and Simpson diversity score

*AL* anastomotic leakage, *CDI Clostridioides difficile*, *OR* odds ratio, *CI* confidence interval, POD postoperative day, *RR* relative risk

### Collagenase-producing bacteria

Bacteria with the ability to produce collagenase were reported in 10 out of 15 studies [34–38, 40, 42, 43, 46, 47]. One study found collagenase-producing bacteria in 14 of 19 (73.7%) bacterial cultures, but it is not specified which bacteria were found [34]. *Enterococcus faecalis* was most frequently reported [34, 40, 43, 46, 47]. On the genus level, there were reports of *Pseudomonas* spp. [38, 42], *Klebsiella* spp. [38, 42], and *Proteus* spp. [36] in samples from AL patients. On the species level, both *Pseudomonas aeruginosa* [47] and *Klebsiella pneumoniae* were found [36] in samples from AL patients. Two studies had CDI as the primary outcome [35, 37]. One study did not find *Enterococcus* spp. among the AL patients, although some of the control patients without AL had *Enterococcus* spp. in the drain fluid [36]. None of the studies presented information of the actual presence of collagenase in the bacterial samples.

### Risk of bias

The Newcastle Ottawa scale was used for all studies, as no randomized trials were included. The median score for all studies was six stars (range 3–7). For the cohort studies, the score was five stars (range 4–6), whereas the case–control studies had a median score of six stars (range 3–7). Very few stars were assigned in the “Comparability” category, as most studies present a mixture of both colonic and rectal resections without segregating the bacterial findings. Only five studies were given a star in Comparability point 1a [36–38, 46, 48], and just one study in Comparability point 1b [35]. Details of the bias assessment are presented in Table 4.

**Table 4:** Bias assessment

Author	**Selection**				**Comparability**		**Exposure/outcome**			**Bias**^**a**^
	**1**	**2**	**3**	**4**	**1a**	**1b**	**1**	**2**	**3**	
Anderson et al. [34]			*				*	*	*	4
Baker et al. [35]				*		*	*	*	*	5
Bilgin et al. [36]	*	*		*	*		*	*	*	7
Calu et al. [37]	*		*		*		*	*	*	6
Fouda et al. [38]	*	*		*	*		*	*	*	7
Jin et al. [39]	*			*			*	*	*	5
Komen et al. [40]	*	*		*			*	*	*	6
Li et al. [41]	*			*			*	*	*	5
Lohsiriwat et al. [42]	*		*				*	*	*	5
Mima et al. [43]	*	*		*			*	*	*	6
Palmisano et al. [44]				*			*	*		3
Reuvers et al. [45]	*	*		*			*	*	*	6
Sparreboom et al. [46]	*	*		*	*		*	*	*	7
Tominaga et al. [47]		*		*			*	*	*	5
van Praagh et al. [48]	*	*		*	*		*	*	*	7

^a^According to the Newcastle–Ottawa scale

## Discussion

This systematic review provides an overview of the bacteria associated with AL after colorectal surgery. The main finding in our study is that 10 out of 15 studies identify one or several bacteria among AL patients that have the potential to produce collagenase(s), although no studies had analyzed the collagenolytic activity of the samples. The studies varied greatly with regard to sample material, study method, and time of sample collection.

The most commonly found bacteria in AL was *Enterococcus faecalis*, from studies using culture or qPCR as study method. This is a common bacteria in the intestinal tract, as it (together with *Enterococcus faecium*) constitutes up to 1% of the adult gut microbiota [50]. Under normal circumstances, it is not a cause of infection, but in case of dysbiosis in the gut (for instance after bowel preparation, antibiotics, and surgical stress), it can begin to express virulence factors that make it a cause of infection. Two studies reported that *Clostridioides difficile* (transferred from the genus *Clostridium* in 2016) infection implied an increased risk of AL with an odds ratio of 2.39 [35] and a relative risk of 13.7 [37]. *C. difficile* is also a common gut bacteria; it is most common among infants and decreases with age [51]. Infections with *C. difficile* usually occur during hospital admissions and antibiotic treatment. *C. difficile* can cause sepsis and produce toxins capable of causing gastrointestinal symptoms varying from mild diarrhea to pseudomembranous enterocolitis and toxic megacolon. Patients who received neoadjuvant chemoradiotherapy have an increased risk of developing CDI [52] and severe dysbiosis of the gut microbiota [53].

*Pseudomonas* spp. (including *Pseudomonas aeruginosa*) were found in several studies. There are conflicting reports in the literature as to whether *P. aeruginosa* is an ordinary member of the normal gut microbiota or not; some argue that this is the case [54, 55], whereas others state otherwise [56, 57]. Treatment of *P. aeruginosa* is usually challenging because the bacteria is resistant to several types of antibiotics [58]. *Klebsiella pneumoniae* is also part of the normal gut microbiota and is a common cause of nosocomial infection. *Klebsiella pneumoniae* and *Pseudomonas aeruginosa* have approximately 99% similar DNA sequences, which makes them susceptible to being mistaken for one or another when interpreting results from microbiome studies [59]. Another genus of bacteria in the gut is *Bacteroides*, and several species can produce collagenase [60]. There are more than 20 known species, most of which can be isolated from human feces. In the studies in this review, *Bacteroides* spp. were overrepresented among AL patients.

Most of the studies used either culture, qPCR, or *C. difficile* immunoassay to identify the bacteria, and all of these studies detected one or more collagenase-producing bacteria. All the qPCR studies targeted *E. faecalis* and *Escherichia coli* [40, 43, 46], and one study also targeted the *Bifidobacterium* genus and *Fusobacterium nucleatum* [43]. All these species are known to be able to produce collagenase.

The five studies that used DNA sequencing methods did find bacterial differences between the AL and non-AL groups. Still, none of the reported bacteria are known to be capable of producing collagenase. This could be due to underreporting of the results, as microbiome studies generate vast amounts of output data, and a complete presentation of the data on the species level rarely seems appropriate. One significant advantage of microbiome analyses is that the anaerobic bacteria are not eliminated by the sampling method, as might be the case using the conventional culture because sampling these bacteria alive requires special attention and equipment [61].

In the majority of the studies, bacteria with the capability to produce collagenase were found among AL patients, and to a lesser degree among patients without AL. According to the literature, all of these bacterial species are known as common members of the intestinal microbiota. This implies that it is not unusual for both patients with or without AL to have these bacteria in the intestine before surgery. However, it is noteworthy that once AL occurs, bacteria with the capability to produce collagenase are a frequent finding in the surroundings of the anastomosis. It is impossible to know whether this is the cause of AL, but it does show some degree of association between this selected group of bacteria and AL. The production of collagenase in a bacterial sample could be quantified by means of zymography [62], or transcriptomic analysis to assess gene activity. Other potential pathways for bacteria to contribute to the development of AL could be that their presence induces a cascade of reactions in the intestine after surgery. Animal models have shown that *E. faecalis* can activate two alternative pathways that lead to collagen degradation. One is through activation of human recombinant MMP9 [18], the other through activation of tissue plasminogen to active plasmin that can cleave collagen and also active MMPs [63]. The functional pathways of the bacteria are outside the scope of this review.

The review has identified some challenges/limitations originating from the current literature. It contains a limited number of studies with a relatively small number of patients in each study, although one study had more than 46,000 patients [35]. All studies have different ways of identifying and reporting bacterial findings, making them very hard to compare equally. Several studies did not report basic confounders for AL, such as smoker status, body mass index, ASA classification, renal disease, neoadjuvant chemoradiotherapy, or blood loss during surgery. Only a minor part of the studies had reports of bowel preparation, preoperative antibiotics, anastomotic technique, and the use of a diverting ileostomy; factors that might also be important to the overall quality of the surgery.

All studies are based on retrospective data, and there might be a degree of reporting bias regarding which results are included in the papers. Only a selection of the bacterial findings may be described in the papers. This could be due to various reasons, for instance, that the complete findings would take up too much space in the papers, or the authors decided that some of the information was irrelevant. In addition, there might be a degree of underreporting of complications in either the patient charts or database data.

The strengths of this review are that it follows PRISMA guidelines and was registered at PROSPERO before the literature search. This increases the transparency in the review process and reduce selection bias. The review is, to our knowledge, the first that aims at describing the microbiology in the surroundings of an AL, based on all available clinical studies. Even though narrative reviews on the association between bacteria in the gut and AL have been published [64–69], this is the first systematic review using multiple literature sources, and the first to focus on bacteria with collagenase-producing capability.

The amount of gut microbiome studies increases yearly, as both analytical capacity and availability increase, and costs are constantly reduced. To increase the quality of microbiome studies, the STORMS (Strengthening The Organization and Reporting of Microbiome Studies) checklist was formulated in 2021 [70]. None of the microbiome studies presented in this review used the STORMS checklist. However, three studies [41, 44, 48] were published before the STORMS checklist.

In conclusion, we found that collagenase-producing bacteria were more common in patients with AL following colorectal surgery than in patients without AL, but the significance is unclear. Future studies could focus on the pathogenicity of bacteria found in AL after colorectal surgery.

### Supplementary Information

Below is the link to the electronic supplementary material.

Supplementary file1 (PDF 507 KB)

Supplementary file2 (PDF 455 KB)

Supplementary file3 (PDF 411 KB)

## Data Availability

All data generated or analyzed in the study are included in this article. Further inquiries can be directed to the corresponding author.
