# Supplementary material for: Collagenase-producing bacteria are common in anastomotic leakage after colorectal surgery: a systematic review
Source: Int J Colorectal Dis. 2023 Dec 1;38(1):275. doi: 10.1007/s00384-023-04562-y (PMC10692267; doi:10.1007/s00384-023-04562-y)
Supplement: Supplementary file 1 — Supplementary file1 (PDF 507 KB) [file 384_2023_4562_MOESM1_ESM.pdf]

Online Resource 1

Collagenase-producing bacteria are common in anastomotic leakage after colorectal surgery: a systematic review

Journal name: International Journal of Colorectal Disease

Anders Bech Jørgensen[1,4], MD (ORCiD 0000-0002-7385-0511)

Isabella Jonsson[1], MD

Lennart Friis-Hansen[2,3,4], MD, DMSc (ORCiD 0000-0002-7222-0163)

Birgitte Brandstrup[1,4], MD, PhD (ORCiD 0000-0003-2659-1198)

[1]Department of Surgery, Holbæk Hospital, part of Copenhagen University Hospitals, Region Zealand, Denmark

[2]Department of Clinical Biochemistry, Bispebjerg and Frederiksberg University Hospitals, Capital Region, Denmark

[3]Department of Microbiology, Rigshospitalet, Capital Region, Denmark

[4]Department of Clinical Medicine, Faculty of Health, University of Copenhagen

Correspondence

Anders Bech Jørgensen

Department of Surgery

Holbæk Hospital, part of Copenhagen University Hospitals

Smedelundsgade 60

4300 Holbæk

Denmark

E-mail: dr.bech@gmail.com

Online Resource 1: Overview of bacteria with the ability to produce collagenase

**Bacterial species**	**Collagenase**					**Gelatinase**	**Gut**
	**Type I**	**Type IV**	**Other**	**DNA**	**Clinical**		
Aggregatibacter actinomycetemcomitans[a] [1,2]	X	X					
Bacillus alvei [3]	X					X	
Bacillus cereus [4–7]	X	X	FALGPA			X	X
Bacillus licheniformis [8]	X					X	
Bacillus pumilus [9]	X					X	
Bacillus subtilis [10,11]	X					X	X
Bacteroides asaccharolyticus [2,12,13]	X	X			X	X	X
Bacteroides bivius [14]	X					X	X
Bacteroides capillus [1]	X						X
Bacteroides corporis [12]	X						X
Bacteroides denticola [12]	X						X
Bacteroides distasonis [14]	X						X
Bacteroides endondontalis [2]		X					X
Bacteroides fragilis [1,15]	X			X[c]		X	X
Bacteroides gingivalis [1,2,12]	X	X					X
Bacteroides intermedius [1,2,12]	X	X					X
Bacteroides levii [12]	X						X
Bacteroides loescheii [12]	X						X
Bacteroides melaninogenicus [1,5,13,14,16]	X				X	X	X
Bacteroides oris [1]	X						X
Bacteroides thetaiotaomicron [1,14]	X						X
Bacteroides spp. [17]	X					X	X
Bacteroides vulgatus [13]	X				X	X	X
Bifidobacterium spp. [17]	X					X	X
Brucella melitensis [14]	X						
Clostridium difficile [13,18,19]	X		Type III[d]		X	X	X
Clostridium histolyticum [5,20,21]	X						X
Clostridium perfringens [13,22,23]				X	X	X	
Elizabethkingia meningoseptica[b] [24]	X						
Enterococcus faecalis [4,25–27]	X	X	FALGPA		X	X	X
Eschericia coli [4,14]	X		FALGPA				X
Eubacterium alactolyticum [17]	X						
Fusobacterium nucleatum [2,14]	X	X					X
Klebsiella pneumoniae [7]	X					X	X
Mycobacterium tuberculosis [28]	X						
Peptococcus magnus [13,17,29,30]	X				X	X	
Peptococcus spp. [14]	X					X	
Peptoniphilus asaccharolyticus [14]	X						
Peptostreptococcus anaerobius [13,14]	X				X	X	X
Peptostreptococcus micros [17]	X						
Porphyromonas gingivalis [31–35]	X	X	Type III + V	X		X	X
Proteus mirabilis [14,25,26]	X	X			X	X	X
Pseudomonas spp. [36]	X					X	X
Pseudomonas aeruginosa [14,25,37–39]	X	X			X	X	X
Pseudomonas marinoglutinosa [40]	X					X	
Serratia marcescens [25,41,42]	X	X			X	X	X
Staphylococcus aureus [14]	X					X	X
Staphylococcus epidermidis [14]	X					X	
Stenotrophomonas maltophilia [25]					X		X
Streptococcus agalactiae [4,43]			FALGPA			X	X
Streptococcus mutans [4,44,45]	X		FALGPA, PZ-PLGPA			X	
Streptomyces spp.[46,47]	X		Type III			X	X
Vibrio alginolyticus[e] [48–50]	X			X		X	
Vibrio vulnificus [51,52]	X				X		
Vibrio parahaemolyticus [53]				X			

Unless specified otherwise, all in vitro diagnostics were performed on type I collagen fibers (bovine/calf skin/rat tail). The 'DNA' column denotes bacteria with identified collagenase genes. The 'Clinical' column denotes bacteria where collagenase activity was prove in clinical samples. The 'Gelatinase' column denotes reported gelatinase activity, and the 'Gut' column denotes bacteria primarily found in the intestine.
FALGPA N-(3-[2-furyl]acryloyl)-L-leucylglycyl-L-prolyl-L-alanine is a collagenase-specific substrate, *PZ-PLGPA* phenylazobenzyl-oxycarbonyl-L-leucylglycyl-L-prolyl-D-arginine, a collagenase-substrate.
[a]Previously known as *Actinobacillus actinomycetemcomitans*, [b]Previously known as *Flavobacterium meningosepticum*, [c]Sequence comparisons showed close identity to matrix metalloproteases (e.g., human fibroblast collagenase), [d]Origin not specified, [e]Previously known as *Achromobacter iophagus*

References

1. Robertson PB, Lantz M, Marucha PT et al. (1982) Collagenolytic activity associated with Bacteroides species and Actinobacillus actinomycetemcomitans. J Periodontal Res 17(3):275–83. https://doi.org/10.1111/J.1600-0765.1982.TB01154.X
2. Uitto V -J, Haapasalo M, Laakso T et al. (1988) Degradation of basement membrane collagen by proteases from some anaerobic oral micro-organisms. Oral Microbiol Immunol 3(3):97–102. https://doi.org/10.1111/j.1399-302x.1988.tb00092.x
3. Kawahara H, Kusumoto M, Obata H (1993) Isolation and Characterization of a New Type of Collagenase Producing Bacterium, Bacillus alvei DC-1. Biosci Biotechnol Biochem 57(8):1372–3. https://doi.org/10.1271/bbb.57.1372
4. Jackson RJ, Lien Dao M, Lim D V. (1995) Modified FALGPA assay for cell-associated collagenolytic activity. J Microbiol Methods 21(2):209–15. https://doi.org/10.1016/0167-7012(94)00050-H
5. Loesche WJ, Paunio KU, Woolfolk MP et al. (1974) Collagenolytic activity of dental plaque associated with periodontal pathology. Infect Immun 9(2):329–36. https://doi.org/10.1128/iai.9.2.329-336.1974
6. Makinen KK, Makinen PL (1987) Purification and properties of an extracellular collagenolytic protease produced by the human oral bacterium Bacillus cereus (strain Soc 67). J Biol Chem 262(26):12488–95. https://doi.org/10.1016/s0021-9258(18)45232-5
7. Suphatharaprateep W, Cheirsilp B, Jongjareonrak A (2011) Production and properties of two collagenases from bacteria and their application for collagen extraction. N Biotechnol 28(6):649–55. https://doi.org/10.1016/j.nbt.2011.04.003
8. Asdornnithee S, Akiyama K, Sasaki T et al. (1994) Isolation and characterization of a collagenolytic enzyme from Bacillus licheniformis N22. J Ferment Bioeng 78(4):283–7. https://doi.org/10.1016/0922-338X(94)90358-1
9. Wu Q, Li C, Li C et al. (2010) Purification and characterization of a novel collagenase from Bacillus pumilus Col-J. Appl Biochem Biotechnol 160(1):129–39. https://doi.org/10.1007/s12010-009-8673-1
10. Nagano H, To KA (2000) Purification of collagenase and specificity of its related enzyme from Bacillus subtilis FS-2. Biosci Biotechnol Biochem 64(1):181–3. https://doi.org/10.1271/bbb.64.181
11. Tran LH, Nagano H (2002) Isolation and Characteristics of Bacillus subtilis CN2 and its Collagenase Production. J Food Sci 67(3):1184–7. https://doi.org/10.1111/j.1365-2621.2002.tb09474.x
12. Jin KC, Barua PK, Zambon J et al. (1989) Proteolytic activity in black-pigmented bacteroides species. J Endod 15(10):463–7. https://doi.org/10.1016/S0099-2399(89)80025-1
13. Steffen EK, Hentges DJ (1981) Hydrolytic enzymes of anaerobic bacteria isolated from human infections. J Clin Microbiol 14(2):153–6. https://doi.org/10.1128/jcm.14.2.153-156.1981
14. McGregor JA, Lawellin D, Franco-Buff A et al. (1986) Protease production by microorganisms associated with reproductive tract infection. Am J Obstet Gynecol 154(1):109–14. https://doi.org/10.1016/0002-9378(86)90404-7
15. Moncrief JS, Obiso R, Barroso LA et al. (1995) The enterotoxin of Bacteroides fragilis is a metalloprotease. Infect Immun 63(1):175–81. https://doi.org/10.1128/iai.63.1.175-181.1995
16. Hausmann E, Kaufman E (1969) Collagenase activity in a particulate fraction from Bacteroides melaninogenicus. Biochim Biophys Acta 194(2):612–5. https://doi.org/10.1016/0005-2795(69)90127-5
17. Keudell K, Conte M (1976) Enzyme of microbial isolates from infected pulp chambers--a preliminary report. J Endod 2(7):217–9. https://doi.org/10.1016/s0099-2399(76)80137-9
18. Poilane I, Karjalainen T, Barc MC et al. (1998) Protease activity of Clostridium difficile strains. Can J Microbiol 44(2):157–61.
19. Seddon S V, Hemingway I, Borriello SP (1990) Hydrolytic enzyme production by Clostridium difficile and its relationship to toxin production and virulence in the hamster model. J Med Microbiol 31(3):169–74. https://doi.org/10.1099/00222615-31-3-169
20. Bond MD, Van Wart HE (1984) Purification and separation of individual collagenases of Clostridium histolyticum using red dye ligand chromatography. Biochemistry 23(13):3077–85. https://doi.org/10.1021/bi00308a035
21. Jung CM, Matsushita O, Katayama S et al. (1999) Identification of metal ligands in the Clostridium histolyticum ColH collagenase. J Bacteriol 181(9):2816–22. https://doi.org/10.1128/jb.181.9.2816-2822.1999
22. Awad MM, Ellemor DM, Bryant AE et al. (2000) Construction and virulence testing of a collagenase mutant of Clostridium perfringens. Microb Pathog 28(2):107–17. https://doi.org/10.1006/mpat.1999.0328
23. Matsushita O, Yoshihara K, Katayama S et al. (1994) Purification and characterization of Clostridium perfringens 120-kilodalton collagenase and nucleotide sequence of the corresponding gene. J Bacteriol 176(1):149–56. https://doi.org/10.1128/jb.176.1.149-156.1994
24. Grimwood BG, Plummer TH, Tarentino AL (1994) Purification and Characterization of a Neutral Zinc Endopeptidase Secreted by Flavobacterium meningosepticum. Arch Biochem Biophys 311(1):127–32. https://doi.org/10.1006/abbi.1994.1217
25. Guyton KL, Levine ZC, Lowry AC et al. (2019) Identification of Collagenolytic Bacteria in Human Samples: Screening Methods and Clinical Implications for Resolving and Preventing Anastomotic Leaks and Wound Complications. Dis Colon Rectum 62(8):972–9. https://doi.org/10.1097/DCR.0000000000001417

26. Shogan BD, Belogortseva N, Luong PM et al. (2015) Collagen degradation and MMP9 activation by Enterococcus faecalis contribute to intestinal anastomotic leak. Sci Transl Med 7(286):286ra68. https://doi.org/10.1126/scitranslmed.3010658
27. Mäkinen PL, Clewell DB, An F et al. (1989) Purification and substrate specificity of a strongly hydrophobic extracellular metalloendopeptidase (“gelatinase”) from Streptococcus faecalis (strain 0G1-10). J Biol Chem 264(6):3325–34. https://doi.org/10.1016/S0021-9258(18)94069-X
28. Takahashi S (1967) Isolation of a collagenolytic enzyme from Mycobacterium tuberculosis. J Biochem 61(2):258–9. https://doi.org/10.1093/oxfordjournals.jbchem.a128538
29. Krepel CJ, Gohr CM, Edmiston CE et al. (1991) Anaerobic pathogenesis: collagenase production by Peptostreptococcus magnus and its relationship to site of infection. J Infect Dis 163(5):1148–50. https://doi.org/10.1093/infdis/163.5.1148
30. Krepel CJ, Gohr CM, Walker AP et al. (1992) Enzymatically active Peptostreptococcus magnus: Association with site of infection. J Clin Microbiol 30(9):2330–4. https://doi.org/10.1128/jcm.30.9.2330-2334.1992
31. Bedi GS, Williams T (1994) Purification and characterization of a collagen-degrading protease from Porphyromonas gingivalis. J Biol Chem 269(1):599–606. https://doi.org/10.1016/s0021-9258(17)42391-x
32. Houle M-A, Grenier D, Plamondon P et al. (2003) The collagenase activity of Porphyromonas gingivalis is due to Arg-gingipain. FEMS Microbiol Lett 221(2):181–5. https://doi.org/10.1016/S0378-1097(03)00178-2
33. Lawson DA, Meyer TF (1992) Biochemical characterization of Porphyromonas (Bacteroides) gingivalis collagenase. Infect Immun 60(4):1524–9. https://doi.org/10.1128/iai.60.4.1524-1529.1992
34. Odell LJ, Baumgartner JC, Xia T et al. (1999) Survey for collagenase gene prtC in Porphyromonas gingivalis and Porphyromonas endodontalis isolated from endodontic infections. J Endod 25(8):555–8. https://doi.org/10.1016/S0099-2399(99)80379-3
35. Sojar HT, Lee JY, Bedi GS et al. (1993) Purification and characterization of a protease from Porphyromonas gingivalis capable of degrading salt-solubilized collagen. Infect Immun 61(6):2369–76. https://doi.org/10.1128/iai.61.6.2369-2376.1993
36. Hisano T, Abe S, Wakashiro M et al. (1989) Isolation and properties of a collagenase with caseinolytic activity from a Pseudomonas sp. J Ferment Bioeng 68(6):399–403. https://doi.org/10.1016/0922-338X(89)90094-9
37. Diener B, Carrick L, Berk RS (1973) In vivo studies with collagenase from Pseudomonas aeruginosa. Infect Immun 7(2):212–7. https://doi.org/10.1128/iai.7.2.212-217.1973
38. Olivas AD, Shogan BD, Valuckaite V et al. (2012) Intestinal tissues induce an SNP mutation in Pseudomonas aeruginosa that enhances its virulence: possible role in anastomotic leak. PLoS One 7(8):e44326. https://doi.org/10.1371/journal.pone.0044326
39. Schoellmann G, Fisher E (1966) A collagenase from Pseudomonas aeruginosa. Biochim Biophys Acta - Enzymol Biol Oxid 122(3):557–9. https://doi.org/10.1016/0926-6593(66)90052-X
40. Hanada K, Mizutani T, Yamagishi M et al. (1973) The isolation of collagenase and its enzymological and physico-chemical properties. Agric Biol Chem 37(8):1771–81. https://doi.org/10.1080/00021369.1973.10860915
41. Hyoju SK, Klabbers RE, Aaron M et al. (2018) Oral polyphosphate suppresses bacterial collagenase production and prevents anastomotic leak due to Serratia marcescens and Pseudomonas aeruginosa. Ann Surg 267(6):1112. https://doi.org/10.1097/SLA.0000000000002167
42. Molla A, Matsumoto K, Oyamada I et al. (1986) Degradation of protease inhibitors, immunoglobulins, and other serum proteins by Serratia protease and its toxicity to fibroblasts in culture. Infect Immun 53(3):522–9. https://doi.org/10.1128/iai.53.3.522-529.1986
43. Jackson RJ, My Lien Dao, Lim D V. (1994) Cell-associated collagenolytic activity by group B streptococci. Infect Immun 62(12):5647–51. https://doi.org/10.1128/iai.62.12.5647-5651.1994
44. Harrington DJ, Russell RRB (1994) Identification and characterisation of two extracellular proteases of Streptococcus mutans. FEMS Microbiol Lett 121(2):237–41. https://doi.org/10.1111/j.1574-6968.1994.tb07104.x
45. Rosengren L, Winblad B (1976) Proteolytic activity of Streptococcus mutans (GS-5). Oral Surgery, Oral Med Oral Pathol 42(6):801–9. https://doi.org/10.1016/0030-4220(76)90103-1
46. Endo A, Murakawa S, Shimizu H et al. (1987) Purification and properties of collagenase from a Streptomyces species. J Biochem 102(1):163–70. https://doi.org/10.1093/oxfordjournals.jbchem.a122028
47. Petrova D, Derekova A, Vlahov S (2006) Purification and properties of individual collagenases from Streptomyces sp. strain 3B. Folia Microbiol (Praha) 51(2):93–8. https://doi.org/10.1007/BF02932162
48. Heindl MC, Fermandjian S, Keil B (1980) Circular dichroism comparative studies of two bacterial collagenases and thermolysin. Biochim Biophys Acta 624(1):51–9. https://doi.org/10.1016/0005-2795(80)90224-x
49. Takeuchi H, Shibano Y, Morihara K et al. (1992) Structural gene and complete amino acid sequence of Vibrio alginolyticus collagenase. Biochem J 281(Pt 3):703–8. https://doi.org/10.1042/bj2810703
50. Tong NT, Tsugita A, Keil-Dlouha V (1986) Purification and characterization of two high-molecular-mass forms of Achromobacter collagenase. Biochim Biophys Acta (BBA)/Protein Struct Mol 874(3):296–304. https://doi.org/10.1016/0167-4838(86)90028-2
51. Miyoshi N, Shimizu C, Miyoshi SI et al. (1987) Purification and Characterization of Vibrio vulnificus Protease.

Microbiol Immunol 31(1):13–25. https://doi.org/10.1111/j.1348-0421.1987.tb03064.x
52. Smith GC, Merkel JR (1982) Collagenolytic activity of Vibrio vulnificus: Potential contribution to its invasiveness. Infect Immun 35(3):1155–6. https://doi.org/10.1128/iai.35.3.1155-1156.1982
53. Miyoshi SI, Nitanda Y, Fujii K et al. (2008) Differential gene expression and extracellular secretion of the collagenolytic enzymes by the pathogen Vibrio parahaemolyticus. FEMS Microbiol Lett 283(2):176–81. https://doi.org/10.1111/j.1574-6968.2008.01159.
