# Supplementary material for: Collagenase-producing bacteria are common in anastomotic leakage after colorectal surgery: a systematic review
Source: Int J Colorectal Dis. 2023 Dec 1;38(1):275. doi: 10.1007/s00384-023-04562-y (PMC10692267; doi:10.1007/s00384-023-04562-y)
Supplement: Supplementary file 3 — Supplementary file3 (PDF 411 KB) [file 384_2023_4562_MOESM3_ESM.pdf]

Online Resource 3

Collagenase-producing bacteria are common in anastomotic leakage after colorectal surgery: a systematic review

Journal name: International Journal of Colorectal Disease

Anders Bech Jørgensen[1,4], MD (ORCiD 0000-0002-7385-0511)

Isabella Jonsson[1], MD

Lennart Friis-Hansen[2,3,4], MD, DMSc (ORCiD 0000-0002-7222-0163)

Birgitte Brandstrup[1,4], MD, PhD (ORCiD 0000-0003-2659-1198)

[1]Department of Surgery, Holbæk Hospital, part of Copenhagen University Hospitals, Region Zealand, Denmark

[2]Department of Clinical Biochemistry, Bispebjerg and Frederiksberg University Hospitals, Capital Region, Denmark

[3]Department of Microbiology, Rigshospitalet, Capital Region, Denmark

[4]Department of Clinical Medicine, Faculty of Health, University of Copenhagen

Correspondence

Anders Bech Jørgensen

Department of Surgery

Holbæk Hospital, part of Copenhagen University Hospitals

Smedelundsgade 60

4300 Holbæk

Denmark

E-mail: dr.bech@gmail.com

Online Resource 3: Search strategy for Pubmed, EMBASE, Google Scholar and Cochrane CENTRAL

	**Bloc #1**	**Bloc #2**	**Bloc #3**
**Theme**	Anastomotic leakage	Colorectal surgery	Bacterial collagenase
**Keywords**	Anastomotic leakage[Mesh]<br><br>Anastomotic leak*<br>Insufficient anastomosis<br>Anastomotic insufficiency<br>Anastomotic dehiscence<br>Leak*	Colorectal surgery[Mesh]<br><br>Colon<br>Colonic<br>Colorectal<br>Rectal<br>Ileorectal<br>Ileoanal<br>Colorectal surgery<br>Colorectal resection<br>Colorectal anastomosis<br>Colon surgery<br>Colon resection<br>Colon anastomosis<br>Colonic surgery<br>Colonic resection<br>Colonic anastomosis<br>Rectal surgery<br>Rectal resection<br>Rectal anastomosis<br>Ileorectal anastomosis<br>Ileoanal anastomosis	Microbial collagenase[Mesh]<br>Enterococcus[Mesh]<br><br>Collagenase<br>Collagenoly*<br>Bacterial collagenase<br>Microbial collagenase<br>E. faecalis<br>Enterococcus<br>P. aeruginosa<br>Pseudomonas aeruginosa<br>P. mirabilis<br>Proteus mirabilis<br>B. fragilis<br>Bacteroides fragilis<br>Culture<br>16S<br>Sequenc*
**PubMed bloc searches**	"anastomotic leak"[MeSH Terms] OR "anastomotic leak*"[All Fields] OR "insufficient anastomosis"[All Fields] OR "anastomotic insufficiency"[All Fields] OR "anastomotic dehiscence"[All Fields] OR "leak*"[All Fields]	"colorectal surgery"[MeSH Terms] OR "colon"[All Fields] OR "colonic"[All Fields] OR "colorectal"[All Fields] OR "rectal"[All Fields] OR "ileorectal"[All Fields] OR "ileoanal"[All Fields] OR "colorectal surgery"[All Fields] OR "colorectal resection"[All Fields] OR "colorectal anastomosis"[All Fields] OR "colon surgery"[All Fields] OR "colon resection"[All Fields] OR "colon anastomosis"[All Fields] OR "colonic surgery"[All Fields] OR "colonic resection"[All Fields] OR "colonic anastomosis"[All Fields] OR "rectal surgery"[All Fields] OR "rectal resection"[All Fields] OR "rectal anastomosis"[All Fields] OR "ileorectal anastomosis"[All Fields] OR "ileoanal anastomosis"[All Fields]	"microbial collagenase"[MeSH Terms] OR "collagenase"[All Fields] OR "collagenoly*"[All Fields] OR "bacterial collagenase"[All Fields] OR "microbial collagenase"[All Fields] OR "e faecalis"[All Fields] OR "enterococcus"[MeSH Terms] OR "enterococcus"[All Fields] OR "p aeruginosa"[All Fields] OR "Pseudomonas aeruginosa"[All Fields] OR "p mirabilis"[All Fields] OR "Proteus mirabilis"[All Fields] OR "b fragilis"[All Fields] OR "Bacteroides fragilis"[All Fields] OR "16S"[All Fields] OR "culture"[All Fields] OR "sequenc*"[All Fields]
**PubMed**<br>n =216	139,090	545,766	2,999,576
**EMBASE**<br>n = 702	206,708	843,582	4,072,912
**Google Scholar**<br>n = 748	1,040,000	17,800	5,500,000
**Cochrane CENTRAL**<br>n = 10	4,413	48,129	31,896

PubMed search string, 8th of April 2023:

("anastomotic leak"[MeSH Terms] OR "anastomotic leak*"[All Fields] OR "insufficient anastomosis"[All Fields] OR "anastomotic insufficiency"[All Fields] OR "anastomotic dehiscence"[All Fields] OR "leak*"[All Fields]) AND ("colorectal surgery"[MeSH Terms] OR "colon"[All Fields] OR "colonic"[All Fields] OR "colorectal"[All Fields] OR "rectal"[All Fields] OR "ileorectal"[All Fields] OR "ileoanal"[All Fields] OR "colorectal surgery"[All Fields] OR "colorectal resection"[All Fields] OR "colorectal anastomosis"[All Fields] OR "colon surgery"[All Fields] OR "colon resection"[All Fields] OR "colon anastomosis"[All Fields] OR "colonic surgery"[All Fields] OR "colonic resection"[All Fields] OR "colonic anastomosis"[All Fields] OR "rectal surgery"[All Fields] OR "rectal resection"[All Fields] OR "rectal anastomosis"[All Fields] OR "ileorectal anastomosis"[All Fields] OR "ileoanal anastomosis"[All Fields]) AND ("microbial collagenase"[MeSH Terms] OR "collagenase"[All Fields] OR "collagenoly*"[All Fields] OR "bacterial collagenase"[All Fields] OR "microbial collagenase"[All Fields] OR "e faecalis"[All Fields] OR ("enterococcus"[MeSH Terms] OR "enterococcus"[All Fields]) OR "p aeruginosa"[All Fields] OR "Pseudomonas aeruginosa"[All Fields] OR "p mirabilis"[All Fields] OR "Proteus mirabilis"[All Fields] OR "b fragilis"[All Fields] OR "Bacteroides fragilis"[All Fields] OR "16S"[All Fields] OR "culture"[All Fields] OR "sequenc*"[All Fields])

EMBASE search string 8th of April 2023:

(exp 'Anastomotic leakage'/ or 'Anastomotic leak*'.mp. or 'Insufficient anastomosis'.mp. or 'Anastomotic insufficiency'.mp. or 'Anastomotic dehiscence'.mp. or 'Leak*'.mp.) and (exp 'Colorectal surgery'/ or Colon.mp. or Colonic.mp. or Colorectal.mp. or Rectal.mp. or Ileorectal.mp. or Ileoanal.mp. or 'Colorectal surgery'.mp. or 'Colorectal resection'.mp. or 'Colorectal anastomosis'.mp. or 'Colon surgery'.mp. or 'Colon resection'.mp. or 'Colon anastomosis'.mp. or 'Colonic surgery'.mp. or 'Colonic resection'.mp. or 'Colonic anastomosis'.mp. or 'Rectal surgery'.mp. or 'Rectal resection'.mp. or 'Rectal anastomosis'.mp. or 'Ileorectal anastomosis'.mp. or 'Ileoanal anastomosis'.mp.) and (exp 'Microbial collagenase'/ or exp 'enterococcus'/ or Collagenase.mp. or Collagenoly*.mp. or 'Bacterial collagenase'.mp. or 'Microbial collagenase'.mp. or 'E faecalis'.mp. or Enterococcus.mp. or 'P aeruginosa'.mp. or 'Pseudomonas aeruginosa'.mp. or 'P mirabilis'.mp. or 'Proteus mirabilis'.mp. or 'B fragilis'.mp. or 'Bacteroides fragilis'.mp. or 'culture'.mp. or '16s'.mp. or 'Sequenc*'.mp.)

Google Scholar search string, 8th of April 2023:

”Anastomotic leakage”|”insufficient anastomosis”|”anastomotic insufficiency”|”leak*” “colorectal surgery”|“colorectal resection”|“colorectal anastomosis” collagenase|16S|”sequenc*”|culture -blood

Cochrane CENTRAL search, 8th April 2023:

ID	Search	Hits
#1	MeSH descriptor: [Anastomotic Leak] explode all trees	233
#2	“Anastomotic leak*” OR ”insufficient anastomosis” OR ”anastomotic insufficiency” OR ”anastomotic dehiscence” OR "Leak*"	4,413
#3	#1 OR #2	4,413
#4	MeSH descriptor: [Colorectal Surgery] explode all trees	420
#5	"Colon" OR "Colonic" OR "Colorectal" OR "Rectal" OR "Ileorectal" OR "Ileoanal" OR ”Colorectal surgery” OR ”Colorectal resection” OR ”Colorectal anastomosis” OR ”Colon surgery” OR ”Colon resection” OR ”Colon anastomosis” OR ”Colonic surgery” OR ”Colonic resection” OR ”Colonic anastomosis” OR ”Rectal surgery” OR ”Rectal resection ”OR ”Rectal anastomosis” OR ”Ileorectal anastomosis” OR ”Ileoanal anastomosis”	48,129
#6	#4 OR #5	48,129
#7	MeSH descriptor: [Microbial Collagenase] explode all trees	54
#8	MeSH descriptor: [Enterococcus] explode all trees	423
#9	"Collagenase" OR "Collagenoly*" OR ”Bacterial collagenase” OR ”Microbial collagenase” OR ”E. faecalis” OR Enterococcus OR ”P. aeruginosa” OR ”Pseudomonas aeruginosa” OR ”P. mirabilis” OR ”Proteus mirabilis” OR ”B. fragilis” OR ”Bacteroides fragilis” OR "culture" OR "16S" OR "sequenc*"	31,891
#10	#7 OR #8 OR #9	31,896
#11	#3 AND #6 AND #10	95
	Selecting only Cochrane Trials (Cochrane Reviews (n = 82) and Cochrane Protocols (n = 3) excluded), therefore n =	10
